# Generation of Breast Cancer Stem Cells through Epithelial-Mesenchymal Transition

**DOI:** 10.1371/journal.pone.0002888

**Published:** 2008-08-06

**Authors:** Anne-Pierre Morel, Marjory Lièvre, Clémence Thomas, George Hinkal, Stéphane Ansieau, Alain Puisieux

**Affiliations:** 1 Centre Léon Bérard, Lyon, France; 2 Inserm, U590, Lyon, France; 3 Université de Lyon, Lyon1, ISPB, Lyon, France; University of Helsinki, Finland

## Abstract

Recently, two novel concepts have emerged in cancer biology: the role of so-called “cancer stem cells” in tumor initiation, and the involvement of an epithelial-mesenchymal transition (EMT) in the metastatic dissemination of epithelial cancer cells. Using a mammary tumor progression model, we show that cells possessing both stem and tumorigenic characteristics of “cancer stem cells” can be derived from human mammary epithelial cells following the activation of the Ras-MAPK pathway. The acquisition of these stem and tumorigenic characters is driven by EMT induction.

## Introduction

A growing body of evidence supports the notion that only a small subset of cells within a tumor, termed cancer stem cells (CSCs) or tumor-initiating cells, are capable of both tumor initiation and sustaining tumor growth [1]. Two basic arguments underlie the hypothesis that cancer stem cells originate from normal tissue stem cells. First, as tumor development is believed to result from the sequential and progressive accumulation of genetic abnormalities, adult stem cells appear to be ideal initial targets for malignant transformation due to their long lifespans. Second, CSCs share several properties with normal stem cells, such as their capacity for self-renewal and their ability to differentiate [2], [3].

The notion of a stem cell origin of cancer was first introduced in the context of hematological malignancies. This hypothesis has been supported by accumulating evidence in both chronic and acute leukemias [4]–[6]. Additionally, committed hematopoietic progenitor cells, with no inherent self-renewal properties, can be induced to generate cells capable of initiating and maintaining leukemias using leukemogenic fusion proteins [7]–[10], indicating that there is no absolute prerequisite for genetic mutation of normal stem cells.

Over the past few years, candidate cancer stem cells have been identified in a variety of human malignancies including leukemias and a number of solid tumors such as glioblastomas, medulloblastomas and carcinomas [11]–[24]. Breast cancer is the first human carcinoma for which a putative cancer stem cell subpopulation has been isolated [25]. Using *in vitro*-separated tumorigenic cells from malignant human breast cancer-derived pleural effusions, Al Hajj and colleagues isolated a cell population characterized by high CD44 expression and low or undetectable levels of CD24 (CD44^+^CD24^−/low^) [25]. These cells were highly tumorigenic when injected into immunocompromised NOD/SCID mice and shared classic features of normal stem cells, including the capacity for self-renewal and generation of heterogeneous progeny [25]. The stem/progenitor cell phenotype of these cells was further refined by the Daidone group, who were able to grow mammospheres from single-cell suspensions obtained from the dissociation of primary breast tumors [3]. Mammospheres are non-adherent spherical cell clusters obtained in selective culture conditions, that have been shown to be enriched in mammary stem/progenitor cells [26]. The vast majority of cells in culture were CD44^+^CD24^−/low^, and 10 to 20% of these retained the ability to self-renew [3].

Congruent with previously reported experiments using models of hematopoietic malignancies [7]–[10], transformed breast cancer cells were obtained *in vitro* by introducing a series of oncogenes and cancer-associated genes into normal primary human mammary epithelial cells. This experimental system starts with primary human mammary epithelial cells (HMECs), that undergo sequential retroviral-mediated expression of the telomerase catalytic subunit (giving rise to HMEC/hTERT cells), SV40 large T and small t antigens (HMLE cells) and an oncogenic allele of H-Ras, H-Ras^V12^ (HMLER cells) [27]. Using this model, we demonstrate that CD44^+^CD24^−/low^ cells possessing stem-like properties can be generated from CD44^low^CD24^+^ non-tumorigenic mammary epithelial cells through activation of the Ras/MAPK signaling pathway and can be accelerated by EMT induction.

## Results

**Figure 1:**
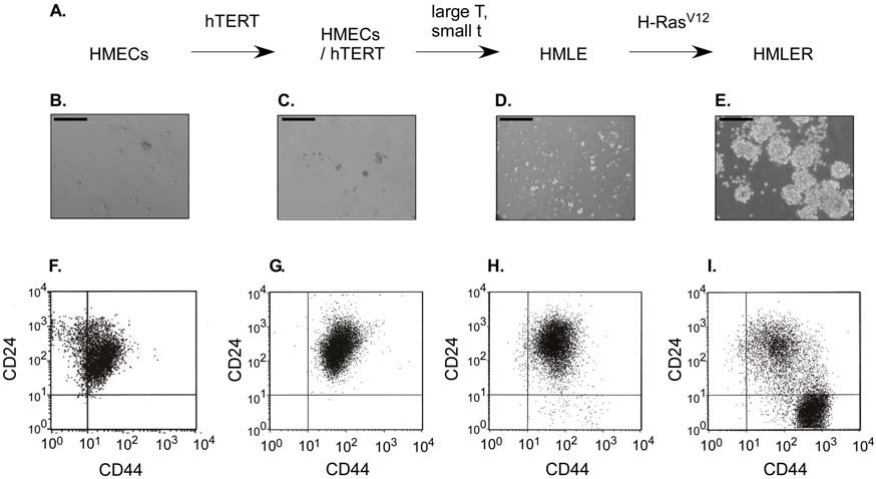
Characterization of the different steps of the *in vitro* model of HMEC transformation. (A) Schematic representation of the successive steps of the transformation. (B,C,D,E) Evaluation of the ability of HMEC-derived cell lines to grow as non-adherent mammospheres. Scale bars = 100 µm. FACS analysis of CD24 and CD44 markers in HMEC-derived cell lines (F,G,H,I). B and F: Primary Human Mammary Epithelial Cell, (HMECs); C and G: hTERT- immortalized HMECs (HME); D and H: HME expressing SV40 small t and large T antigens (HMLE); E and I: H-Ras^V12^-infected HMLE cells (HMLER).

To determine the potential origin of tumorigenic CD44^+^CD24^−/low^ cells, we implemented a model of human breast cancer progression described by Elenbaas *et al*. [27] (Figure 1A). The Weinberg's group showed that HMLER cells (HMECs overexpressing hTERT, SV40 T/t and H-RasV12) were tumorigenic when injected subcutaneously or into the mammary glands of immunocompromised mice, suggesting the possible generation of cancer stem cells. To test this hypothesis, we analyzed the capabilities of the different cell populations (HMECs, HMEC/hTERT, HMLE and HMLER) to grow as non-adherent mammospheres, a property associated with mammary stem/progenitor cells [3], [26]. In contrast with HMECs, HMEC/hTERT and HMLE cells (Figure 1B–D), only HMLER cells grew as non-adherent clusters, suggesting that they displayed both tumorigenic and stem-like properties (Figure 1E). We further analyzed the phenotype of the different HMEC-derived cell lines (expanded in adherent conditions) by fluorescence-activated cell sorting (FACS) using CD44 and CD24 as markers (Figure 1F–I). HMECs and HMEC/hTERT immortalized mammary epithelial cells were CD44^low^CD24^+^ (subsequently referred to as CD24^+^) and CD44^+^CD24^−/low^ cells (subsequently referred to as CD24^−^) were totally undetectable in both cell populations (Figures 1F and 1G). The additional introduction of oncogenes was associated with the appearance and progressive increase of CD24^−^ cells which accounted for 1.4% of HMLE cells and more than 65% of HMLER cells (Figures 1H and 1I). Consistent with the report that several components of the Ras/MAPK pathway are present in the expression profile of CD24^−^ cancer cells [28], this observation suggested that cancer stem cells could be generated in response to the activation of specific signal transduction pathways.

This hypothesis was further investigated by studying using FACS analysis the emergence of CD24^−^ cells following retroviral expression of H-Ras^V12^ in HMLE cells. Whereas this cell population remained low (<2%) in uninfected cells, mutant Ras expression caused its progressive accumulation from 3.2% at day 5 following infection, 10.1% at day 24, 32.1% at day 30, to 65.4% at day 55 (Figure 1I and data not shown). This observation cannot be interpreted as a consequence of an enrichment of a rare subpopulation of cells displaying growth advantage because CD24^−^ and CD24^+^ cells showed similar proliferation potential, as demonstrated by the methylthiazolyldiphenyl-tetrazolium bromide (MTT) assay (data not shown). Of note, the length of time necessary for the emergence of CD24^−^ cells following H-Ras^V12^ introduction is consistent with the initial observation by Elenbaas and colleagues that the tumorigenicity of HMLER cells was associated with the occurrence of secondary events [27]. In order to determine the origin of CD24^−^ cells, we performed cell sorting experiments and single-cell cloning assays following retroviral expression of H-Ras^V12^ in HMLE cells. After sorting of CD24^−^ and CD24^+^ populations, CD24^+^ cells were seeded onto 96-well plates under limiting dilution cloning conditions in order to isolate single cells (day 30 post-infection). After three weeks of cell growth, 35 independent individual clones were isolated and characterized. Whereas 47% of the clones were fully composed of CD24^+^ cells, 33% displayed a heterogeneous population of CD24^+^ and CD24^−^ cells, and 19% were homogeneous for CD24^−^ cells. These observations demonstrate that CD24^−^ cells can originate from CD24^+^ cells, since a single CD24^+^ cell is able to generate either heterogeneous CD24^+^/CD24^−^ clones or homogeneous CD24^−^ clones.

**Figure 2:**
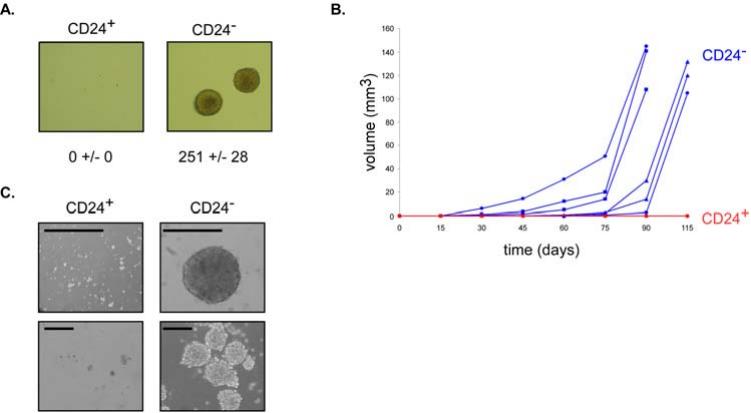
CD24^−^ cells display tumorigenic and stem-like properties. (A) Colony assay. Growth in soft agar. Number of colonies are indicated for 5×10^3^ plated cells (+/− standard deviation, n = 3, 40x magnification). (B) Tumorigenicity assay. Athymic *nude* mice received a single injection of 10^6^ CD24^+^ or CD24^−^ cells to a mammary fat pad. Tumor growth was monitored every 3 days and volume measured every 15 days. Blue lines indicate CD24^−^ cells and red lines indicate CD24^+^ cells, marker shapes represent duplicate injections in separate animals from three independent clones for both cell populations. (C) In contrast to CD24^+^ cells, CD24^−^ cells formed mammospheres in low-adherent conditions. Scale bars = 100 µm.

We next evaluated the transformation and stem-like properties of CD24^+^ and CD24^−^ cells generated by retroviral expression of H-Ras^V12^ in HMLE cells. Unlike CD24^+^ cells, CD24^−^ cells were able to grow in soft agar, a characteristic of transformed cells (Figure 2A). The CD24^−^ population was also able to form tumors when injected into the mammary fat pads of *nude* mice (6/6 for CD24^−^ *versus* 0/6 for CD24^+^ cells, Figure 2B). This difference was shown to be independent of the level of expressed Ras (data not shown). Additionally, in non-adherent conditions, only CD24^−^ cells generated mammospheres, further demonstrating the stem-like properties of HMLER cells (Figure 2C). Of note, similar results were invariably obtained using either the sorted CD24^−^ cell population or CD24^−^ isolated clones (data not shown). Primary spheres obtained from CD24^−^ cells could be enzymatically dissociated with trypsin into single cells to give rise to secondary spheres (data not shown).

**Figure 3:**
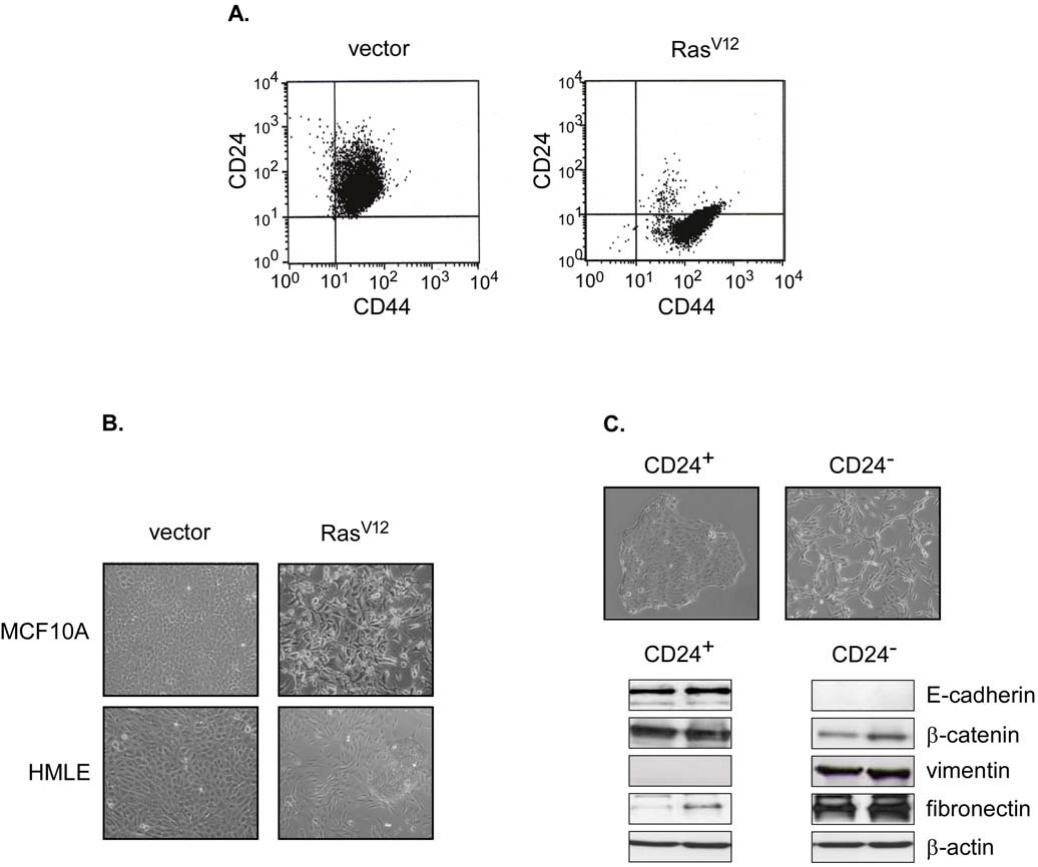
Oncogenic versions of Ras promote a CD24^+^ to CD24^−^ transition and EMT in MCF10A and HMLE cell lines. (A) FACS analysis of CD24 and CD44 markers in MCF10A cells infected either with a K-Ras^V12^ retroviral construct or the corresponding empty vector as a control. (B) Cell morphology of MCF10A cells infected either with a K-Ras^V12^ retroviral construct or the corresponding empty vector as a control (upper panel) and HMLE infected either with a H-Ras^V12^ retroviral construct or the corresponding empty vector as a control (lower panel). Images shown at 40x magnification. (C) CD24^+^ and CD24^−^ display epithelial and mesenchymal features, respectively. Top: Cell morphology. Bottom: Expression analysis as assessed by western-blotting of epithelial (E-cadherin, β-catenin) and mesenchymal (vimentin, fibronectin) markers in two independent clones for both cell populations. Images shown at 40x magnification.

**Figure 4:**
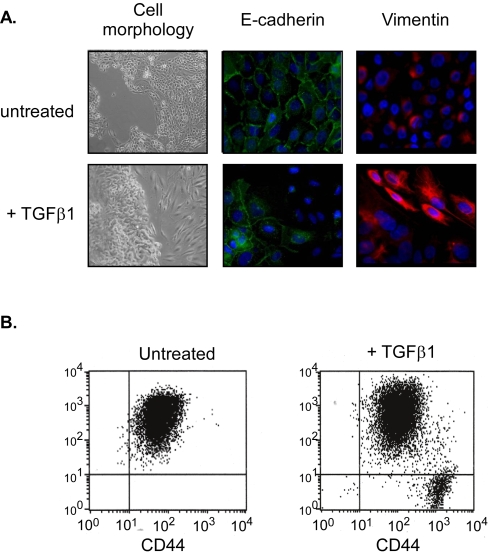
TGFβ1 concomitantly promotes EMT and the CD24^+^ to CD24^−^ transition in CD24^+^ HMLER cells. (A) Treatment of CD24^+^ HMLER with TGFβ1 induces EMT, as assessed by morphology changes as well as loss of E-cadherin (epithelial marker) and induction of vimentin (mesenchymal marker), as assessed by immunofluorescence staining. Images shown at 40x magnification. (B) Treatment of CD24^+^ HMLER with TGFβ1 induces CD24^+^ to CD24^−^ transition. FACS analysis of CD24 and CD44 markers in CD24^+^ cells, untreated or treated with TGFβ1.

**Figure 5:**
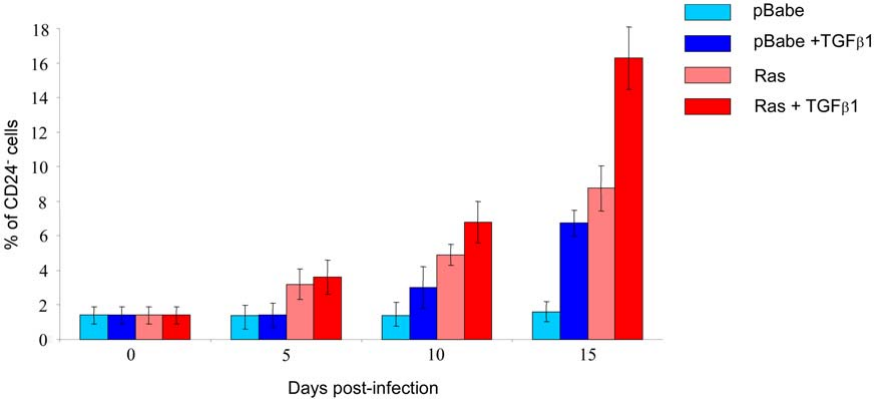
Oncogenic Ras and TGFβ1 cooperate to promote the CD24^+^ to CD24^−^ cells. HMLE cells were infected with an H-Ras^V12^ retroviral expression construct or the empty vector (pBabe) as a control. Two days post-infection, experimental cells were treated with TGFβ1. Percentage of CD24^−^ cells was assessed at different times following infection. Error bars indicate +/− standard deviation of triplicates.

We next attempted to validate our observations in a different cellular context using the immortal human mammary epithelial cell line MCF10A. As we demonstrated in transformed HMECs, MCF10A infection with K-Ras^V12^ [29], was associated with the emergence of CD24^−^ cells: 1% in MCF10A cells infected with empty retroviral vector as compared to 90% in those infected with the K-Ras^V12^ expressing construct (Figure 3A). Intriguingly, enrichment in CD24^−^ cells in both HMLER and MCF10A cell lines was coincided with a morphological change in the cells from a general epithelial appearance to a dominant population of spindle cells, characteristic of mesenchymal cells (Figure 3B). To examine whether CD24^−^ cells might emerge through an EMT process, a morphogenetic process in which cells lose their epithelial characteristics and gain mesenchymal properties, the expression levels of epithelial and mesenchymal markers in both CD24^+^ and CD24^−^ cells, was assessed. In contrast to CD24^+^ cells, CD24^−^ cells, either sorted from the bulk population or cloned, expressed low or undetectable levels of epithelial markers (E-cadherin and β-catenin) and high levels of mesenchymal markers (vimentin and fibronectin), suggesting that they underwent an EMT (Figure 3C). To evaluate such a possibility, we thus examined whether the treatment of HMLER CD24^+^ with TGFβ1, a potent inducer of EMT, led to CD24^−^ cell appearance. Indeed, eight days after treatment, a concomitant enrichment of mesenchymal cells (characterized by a loss of the E-Cadherin and the induction of vimentin expression) and CD24^−^ cells could be observed, confirming our hypothesis (Figure 4). As Ras and TGFβ are known to cooperate in EMT induction, we then assumed that, in presence of TGFβ, the length of time needed to generate CD24^−^ cells from CD24^+^ HMLE cells following infection with Ras should be shortened. As shown in Figure 5, in agreement with our hypothesis, the addition of TGFβ1 significantly accelerated the emergence of CD24^−^ cells from CD24^+^ HMLE cells infected with H-Ras^V12^.

## Discussion

The present work demonstrates that tumorigenic CD44^+^CD24^−/low^ (CD24^−^) cells can originate from primary CD44^low^CD24^+^ (CD24^+^) human mammary epithelial cells (HMECs) following their transformation with a limited number of oncogenes and cancer-associated genes. Specifically, activation of the Ras signaling pathway appears to be a crucial event to facilitate the emergence of CD24^−^ cells. Strikingly, in both HMECs and MCF10A cells, the CD24^−^ phenotype was constantly associated with features of an epithelial-mesenchymal transition (EMT), including the loss of epithelial markers and the concomitant gain of mesenchymal markers. We then assumed that CD24^−^ cells could arise from CD24^+^ through an EMT trans-differentiation process. Accordingly, we showed a cooperative effect of TGFβ and Ras activation as treatment of Ras infected cells with TGFβ1 accelerates the emergence of CD24^−^ cells. These findings are consistent with the recent observation that CD24^−^ cells isolated from breast cancer tissues display a mesenchymal phenotype attributable to the activation of TGFβ and Wnt signaling [30], two pathways known to be involved in EMT [31].

EMT, which was first recognized as a crucial feature of embryogenesis, converts epithelial cells into mesenchymal cells through profound disruption of cell-cell junctions and extensive reorganization of the actin cytoskeleton [32]. Although still controversial, this process is presumed to be required for tumor invasion and metastasis of carcinoma cells by promoting loss of contact inhibition, increased cell motility and enhanced invasiveness [33]. EMT is believed to be governed by complex networks largely influenced by signals from the neoplastic microenvironment. Indeed, *in vitro*, a variety of cytokines, including TGFβ and growth factors like hepatocyte growth factor (HGF), epidermal growth factor (EGF) or fibroblast growth factors (FGFs), can trigger EMT after activation of their cognate receptors in specific cell types. Of note, growth factors transduce signals through the activation of their cognate receptor tyrosine kinases and of their central downstream effector Ras, giving a rationale for the cooperative effect of Ras and TGFβ in EMT promotion [34], [35]. In the experimental model of breast cancer progression, the introduction of an activated version of Ras constitutes the initial event that sensitizes mammary epithelial cells to EMT. However, the delay for EMT induction and the associated emergence of H-Ras^V12^-transformed CD24^−^ cells suggests that additional events are required. These additional events may depend upon environmental EMT-inducing signals since the addition of TGFβ to the culture medium significantly decreases the time required for completing the process and increases the percentage of CD24^−^ cells. Altogether, our observations support the intriguing hypothesis that the CD44^+^CD24^−/low^ cells (or at least a fraction of them) present within a primary breast cancer might reflect the propensity of malignant cells to undergo transdifferentiation and metastasize.

Considering the role of EMT in invasiveness and metastatic dissemination, our observations provide a rational explanation for the prognostic value of the gene-expression signature of CD24^−^ cancer stem cells in breast cancers [28]. This signature, termed IGS (for invasiveness gene signature), has been generated by the Clarke group by comparing the gene-expression profiles of CD24^−^ breast-cancer cells and normal breast epithelium. Importantly, the IGS is significantly associated with both overall survival and metastasis-free survival in patients with breast cancer or with other types of malignancy [28]. Nevertheless, this observation is at odds with the description of cancer stem cells as a minority population within a tumor, because the gene expression profile of these rare cells is likely to be masked when tumors as a whole are analyzed for gene expression [36]. Our findings strongly suggest that the IGS might be a consequence of the oncogenic activation of signaling pathways involved in EMT, invasion and metastasis. This hypothesis is further substantiated by the presence of components of the Ras/MAPK pathway in the IGS, as well as targets of TGFβ, and inducers of EMT and/or mesenchymal markers (MGP, CXCL12, MMP-7, Ets1, Ezrin, Wee1) [28]. It is also highly consistent with the recent observation that breast cancer cell lines containing a high percentage of CD24^−^ cells express basal/mesenchymal markers and display invasive properties [37].

Taken together, our observations demonstrate that, at least *in vitro*, CD44^+^CD24^−/low^ cells can originate from CD44^low^CD24^+^ human mammary epithelial cells after aberrant activation of the Ras/MAPK pathway. They also strongly suggest that the number of CD24^−^ cells within a primary tumor reflects the sensitivity of the cancer cells to EMT-inducing signals. Are these cells potential “tumor initiating cells”? Although not tested in similar experimental conditions, the tumorigenicity of CD24^−^ cells generated in our experimental model appears to be significantly weaker than the one of cancer stem cells originally isolated from human breast cancer-derived malignant pleural effusions by the group of Clarke [25]. On this basis, two (non-exclusive) hypotheses can be proposed. First, in the course of tumor progression, cancer cells with stem-like capabilities can be generated from differentiated pre-malignant cells by acquiring specific genetic alterations. As these somatic abnormalities also provide a growth advantage, the potential number of such cells within a tumor might compensate for their limited stem-like characteristics. Accordingly, these cells could act as an auxiliary power source for tumor progression and metastasis. A second hypothesis is that similar alterations could initially affect normal stem cells, giving rise to genuine cancer stem cells. The Weinberg group recently reported that the tumorigenicity of experimentally transformed mammary epithelial cells is highly dependent upon the cell type of origin [38]. When exposed to microenvironmental signals, these cancer stem cells would display motility capacities due to EMT, lending support to the notion of “mobile cancer stem cells” initially proposed by T. Brabletz [39].

Of note, during the review process of our manuscript, the connection between EMT and stem-like properties has also been strongly supported by the Weinberg laboratory [40]. Using different EMT-inducers, they showed that the induction of EMT in immortalized human mammary epithelial cells is associated with the acquisition of stem-like characteristics. Additionally it was shown that normal, as well as neoplastic breast stem-like cells, express mesenchymal markers. These data further support our findings.

## Materials and Methods

### Cell culture, proliferation and mammosphere-formation assays

Human mammary epithelial cells were provided and cultured as recommended by Lonza. HMEC-derivatives (kindly provided by RA Weinberg) were cultured in 1:1 Dulbecco's Modified Eagle's Medium (DMEM)/HAMF12 medium (Invitrogen) complemented with 10% FBS (Cambrex), 100 U/ml penicillin-streptomycin (Invitrogen), 2 mM L glutamine (Invitrogen), 10 ng/ml human epidermal growth factor (EGF) (PromoCell), 0.5 µg/ml hydrocortisone (Sigma) and 10 µg/ml insulin (Sigma) and treated with 10ng/ml recombinant TGFβ1 (Peprotech) for 15 days.

MCF10A infected with a retroviral K-Ras^V12^ expression construct was generously provided by the Ben-Ho Park's laboratory and maintained in culture as described in [29]. The Phoenix A packaging cell line was maintained as recommended by the ATCC.

For mammosphere formation, after filtration through a 30 µm pore filter, single-cells were plated at 10^5^ cells/ml in Corning 3261 ultra-low attachment culture dishes in the growth medium described above. Primary cell spheres were enzymatically dissociated with 0.05% trypsin for 15 minutes at 37°C to obtain single-cell suspension.

### Retroviral infection

2×10^6^ Phoenix cells were transfected by calcium-phosphate precipitation with 10 µg of retroviral vector pBabe-H-Ras^V12^. 48 hours post-transfection, the supernatant was collected, filtered, supplemented with 4 µg/ml of polybrene (Sigma) and combined with 10^6^ HMLE cells for 3 hours. Infected cells were selected after 48 hours with puromycin (0.5 µg/ml).

### Colony assay

To measure anchorage-independent growth, cells were detached with trypsin and resuspended in growth medium. Plates were prepared with a coating of 0.75% agarose (Cambrex) in growth medium and then overlaid with a suspension of cells in 0.45% agarose (5×10^3^ cells/well). Plates were incubated for 3 weeks at 37°C and colonies were counted under microscope.

### Flow-cytometric analysis

Identification and sorting of CD24^+^ and CD44^+^ cells were performed using monoclonal anti-CD24-PE ML5 and anti-CD44-FITC G44-26 antibodies (PharMingen), a FACScan (Becton Dickinson) and a DIVA instrument (Becton Dickinson).

### Mouse injection

Animal maintenance and experiments were performed in accordance with the animal care guidelines of the European Union and French laws. Six-week old female Athymic *Swiss nude* mice (C. River laboratories) were injected with 10^6^ CD24^−^ or CD24^+^ cells into a fat pad of mammary gland. Tumor growth was monitored twice a week with callipers at the site of injection. Animals were sacrificed as soon as tumor size reached 1.5 cm in diameter.

### Immunoblot analysis

Cells were washed twice with phosphate buffered saline (PBS) containing CaCl_2_ and then lysed in RIPA buffer. Protein expression was examined by western blot using monoclonal anti-E-cadherin clone 36 (Becton Dickinson), anti-β-catenin clone 14 (Becton Dickinson), anti-fibronectin FN-15 (Sigma), anti-vimentin V9 (Dako), anti-β-actin AC-15 (Sigma) antibodies for primary detection. Horseradish peroxidase-conjugated rabbit anti-mouse antibody (Amersham) was used as a secondary antibody. Western-blots were revealed using an ECL detection kit (Amersham).

### Immunofluroescence

About 10^4^ cells were seeded on 4-well Lab-TekII chamber slide. After TGFβ1 treatment, the cells were washed with PBS twice, fixed in 3% parformaldehyde (Sigma) and permeabilized in 0.1% Triton 100X (Sigma) in PBS buffer at 4°C for 30 minutes. The cells were then washed 3 times with PBS and incubated with blocking solution (10% horse serum in PBS). The cells were then incubated with the primary antibodies anti-E-cadherin clone 36 (Becton Dickinson), or anti-vimentin V9 (Dako) overnight at 4°C. The cells were washed 3 times in PBS and incubated with the appropriate secondary antibodies (Dako) for 1 hour at room temperature. Finally the cells were washed 3 times in PBS and incubated with Hoechst (Sigma) for 5 minutes. The slides were washed extensively with PBS and mounted with Fluoromount-G (SouthernBiotech). All matched samples were photographed (control and test) using immunofluorescence microscope and identical exposure times.
